# Dissipation kinetics and exposure of spirotetramat and pymetrozine in open fields, a prelude to risk assessment of green bean consumption

**DOI:** 10.1007/s11356-023-26100-7

**Published:** 2023-03-27

**Authors:** Farag Malhat, Mona Bakery, Osama Abdallah, Mohamed Youssef, Walaa Abd El Ghany, Amira Abdallah, Sarah Greish, Mona M. Gaber, Indra Purnama, Shokr Abdelsalam, Mohamed Tawfic Ahmed

**Affiliations:** 1 Pesticide Residues and Environmental Pollution Department, Central Agricultural Pesticide Laboratory, Agricultural Research Center, Dokki, 12618 Giza Egypt; 2 Chemistry Department, Faculty of Science, Helwan University, Helwan, Egypt; 3 Plant Protection Department, Faculty of Agriculture, Ain Shams University, Cairo, Egypt; 4 Plant Protection Department, Faculty of Agriculture, Suez Canal University, Ismailia, 41522 Egypt; 5 Lancang Kuning University, Pekanbaru, Indonesia

**Keywords:** Green beans, Pymetrozine, Spirotetramat, Method validation, Risk assessment

## Abstract

Determination and dissipation kinetics of pymetrozine and spirotetramat in green bean were studied using a QuEChERS method coupled to high-performance liquid chromatography-tandem mass spectrometry. Pymetrozine recoveries ranged between 88.4–93.7%, with relative standard deviation (RSD) of 5.5–14.4%. For spirotetramat the recoveries ranged between 91.7–103.4%, and the RSD were in the range of 3.2 to 12.4%. The limits of quantification (LOQs) were 0.01 mg/kg and 0.005 mg/kg for pymetrozine and spirotetramat, respectively.

The developed analytical method was used to study the degradation rates of pymetrozine and spirotetramat in green bean grown in open field. Results showed that pymetrozine and spirotetramat followed the first-order kinetics model with half-lives of 3.3 days and 4.2 days, respectively. Furthermore, risk assessment was carried out which showed that, the chronic risk quotient (RQc) values for pymetrozine and spirotetramat were much lower than 100%. The present results indicated that the health risks posed for consumers by the pymetrozine and spirotetramat residues were negligible at the recommended dosages.

## Introduction

Green bean (Phaseolus vulgaris L.) is a common food worldwide which is eaten either raw or cooked. It is one of the major leguminous crops grown in Egypt, the green pods and dry seeds are marketed for local consumption and also for exportation. (Badawy et al. 2020). Egypt's annual production amounts to about 265 thousand tons (FAOSTAT 2020). With such high productivity rate, Egypt ranks tenth among the green bean exporting countries in the world. In addition to the economic value of green beans, it improves the soil quality as the parts of roots remaining in soil after harvesting is considered valuable fertilizers enriching the soil (Fahad et al. 2015a).

The green bean plant is susceptible to a score of insect pests, including aphids, thrips, leaf worms and others that affect both the quality and quantity of the yield, which requires regular insecticides applications. In Egypt, the Agricultural Pesticides Committee (APC) recommends the use of different classes of pesticides on this crop to control various pests and diseases (Agricultural Pesticides Committee 2020). Spirotetramat and pymetrozine pesticides are widely used to control sucking pests in green beans. Even though, these pesticides are beneficial in protecting green beans from pests, they may accumulate in the edible parts causing potential health risk to consumers (Ahmed et al. 2014; Xu et al. 2021).

**Fig. 1:**
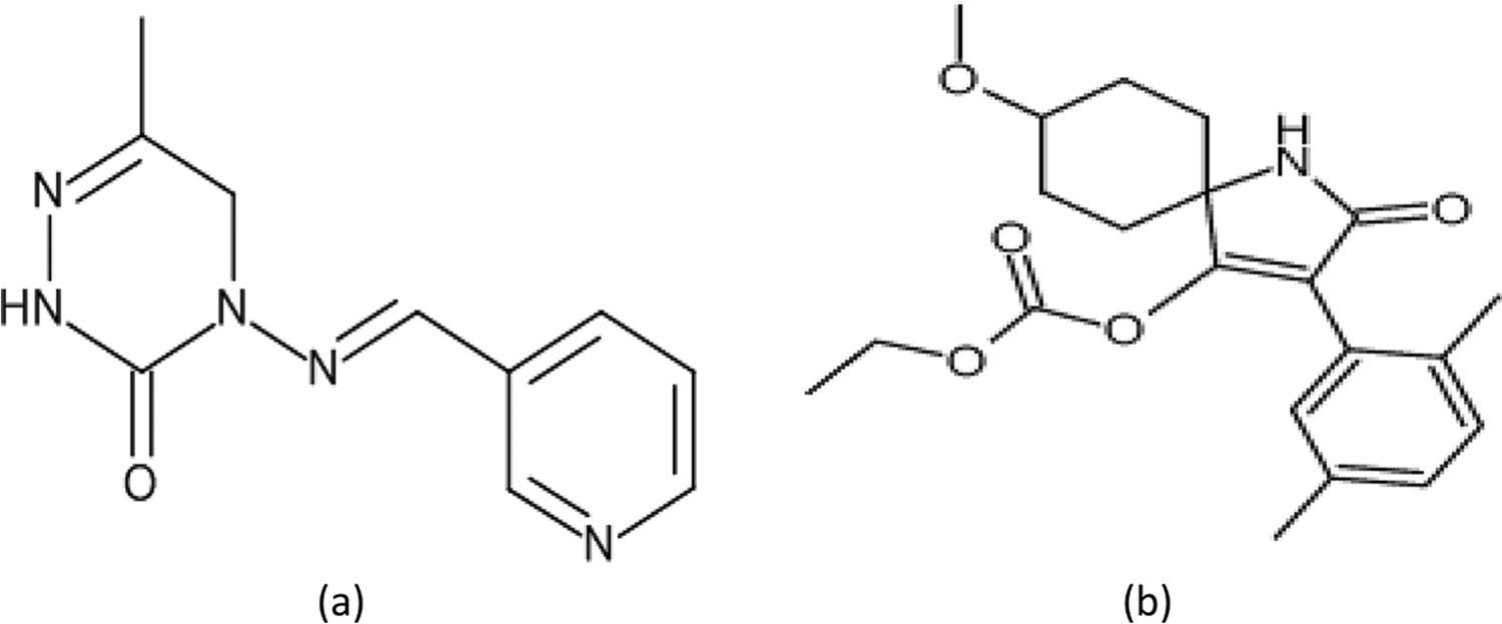
Chemical structure of pymetrozine (**a**) and spirotetramat (**b**)

Spirotetramat, cis-3-(2,5-dimethlyphenyl)-8-methoxy-2-oxo-1-azaspiro (Fig. 1a), belongs to the chemical class of tetramic acid derivative. It is one of the recommended and effective insecticides used to control widespread insects such as aphids and thrips in vegetables and fruits by inhibiting the action of acetyl-CoA carboxylase (Brück et al. 2009; Kay and Herron 2010; Kumar et al. 2009; Kumar and Kuttalam 2009; Smiley et al. 2011).

Pymetrozine, 4,5-dihydro-6-methyl-4-[(3- pyridyl methylene)-amino]-1,2,4-triazin-3(2H)-one (Fig. 1b) is another effective pesticides against sucking insects. It is a pyridine azomethine-based insecticides, that inhibits nerve-muscle interaction of the sucking insects (Lashkari et al. 2007; Li et al. 2011; Shen et al. 2009). The Environmental Protection Agency (EPA) has deemed pymetrozine a "possible" human carcinogen (Zhang et al. 2007). Therefore, it is necessary to monitor the residues of pymetrozine in green beans.

The irrational use of pesticides raised concerns due to their risk to human health. In this context, governments and international organizations are committed to regulate and control the use of pesticides to protect consumers’ health. Setting a maximum acceptable residue limit (MRL) is an integral element of Good Agricultural Practices (Handford et al. 2015). Moreover, a pre-harvest interval period (PHI) has been also introduced as another precautionary measure to protect consumers (MacLachlan and Hamilton 2010).

Several studies were conducted to monitor residue of spirotetramat or pymetrozine in many crops including cotton (Pandiselvi et al. 2010), grapes (Mohapatra et al. 2015; Vemuri et al. 2014), chilli (Chahil et al. 2015), mangoes (Mohapatra et al. 2012b), spinach (Chen et al. 2016), tomatoes (Abd-Alrahman and Kotb 2020; Abd Al-Rahman et al. 2012), strawberry (Xu et al. 2021) and many other fruit and vegetable crops (Han et al. 2013; Jia et al. 2019; Li et al. 2011; Singh et al. 2013). However, to the best of our knowledge no data has been reported about dissipation of spirotetramat and pymetrozine in green beans under field conditions.

To monitor spirotetramat and pymetrozine residues in food commodities different analytical methods were used such as, high performance liquid chromatography (HPLC) (Abd-Alrahman and Kotb 2020; Abd Al-Rahman et al. 2012; Cabizza et al. 2007; Chahil et al. 2015; Hong et al. 2011; Mohapatra et al. 2012a; Pandiselvi et al. 2010; Shen et al. 2009; Singh et al. 2013; Vemuri et al. 2014), ultra-performance liquid chromatography coupled with tandem mass spectrometry (UPLC-MS/MS), liquid chromatography with tandem mass spectrometry (LC–MS/MS) (Chen et al. 2016; Dias et al. 2013; Fernandes et al. 2014; Jia et al. 2019; Li et al. 2011, 2016; Xu et al. 2021; Zhang et al. 2015; Zhu et al. 2013) and gas chromatography mass spectrometry (GC/MS) (Jang et al. 2014; Mohapatra et al. 2015). Considering the extensive use of spirotetramat and pymetrozine in green beans there is an urgent need to develop a quick, precise and accurate method to simultaneously determine them.

This study aimed to develop a simple, accurate, and rapid method for simultaneous quantitation of spirotetramat and pymetrozine residues in green beans using UPLC–MS/MS. A continuous application approach using single and double recommended doses was carried out to investigate the dissipation patterns, residue levels, and risk assessment of spirotetramat and pymetrozine in green beans cultivated in open field conditions at different time intervals. The results could provide guidance for the safe use of spirotetramat and pymetrozine in green beans under open field conditions.

## Materials and methods

### Chemicals and reagents

Certified standard samples of pymetrozine (purity, 99.4%) and spirotetramat (purity, 99.4%) were purchased from ChemService (West Chester, PA, USA). HPLC grade acetonitrile (ACN) and methanol (MeOH), LC–MS grade formic acid, were obtained from Fisher Scientific (Loughborough, UK). Sodium chloride and magnesium sulfate anhydrous were supplied from Chem-Lab NV (Zedelgem, Belgium). Primary secondary amine (PSA, 40–60 μm) was obtained from Agilent Technologies (DE, USA). The formulations of pymetrozine (50%, wettable granule, WG), and spirotetramat (10 %, suspension concentrate, SC) were provided from the local markets.

### Preparation of standard solutions

Standard stock solutions of pymetrozine and spirotetramat (each at 100 mg/L) were prepared by dissolving 10.06 mg of each standard in 100 mL ACN. The intermediate mixture standard solution of 10 mg/L was prepared in ACN by further dilution. Mixed standard working solutions with equal concentrations were serially diluted using ACN to construct calibration curves. All the solutions were stored at 4 °C.

### Field trail

The field trials were carried out, during the growing season 2020, in Giza governorate, located south of Cairo. Green beans were cultivated in February 2020. Two independent experiments were carried out, one for each insecticide, with different plots (50 m^2^) for each insecticide. A buffer zone (15 m^2^) was made to separate adjacent plots to avoid cross-contamination.

Samples were collected 0, 2 h, 1, 3, 7, 10, 14 days post treatment to monitor each insecticide residues in green beans. Samples were immediately transferred to the laboratory, cut into small pieces about 3 cm and then frozen at -20 °C overnight, homogenized the next day using a Hobart food cutter (Hobart Corp., Troy, OH, USA).

### Terminal residues

Pymetrozine and spirotetramat were applied 2 or 3 times each, at two dosage level 100 g a.i/ha (low level) and 200 g a.i/ha (high level) after the edible part of the fruits was formed. Representative samples were collected according to Codex guidelines (FAO and WHO 2019) at several pre-harvest Intervals (PHI). For the assessment of terminal residues, samples were taken at 3, 7 and 14 days after the last treatment.

### Sample extraction

Quick, easy, cheap, effective, rugged, and safe (QuEChERS) is the most common technique used due to their simplicity, good purification efficiency, and low organic solvent consumption (AOAC 1990; Duan et al. 2018; Hong et al. 2011; Lehotay et al. 2005; Lehotay^2007^;Singh et al. 2013; Yang et al. 2015). The QuEChERS method includes an extraction step with acetonitrile (ACN) and partitioning using MgSO_4_. The extraction is cleaned up by primary secondary amine (PSA), octadecyl modified silica (C18), and graphite carbon black (GCB) dispersive solid phase extraction (Anastassiades et al. 2003b, 2003a).

The frozen homogenized green bean (10 g) was weighed into a 50 mL polypropylene centrifuge tube, and 10 mL ACN was added to it. Samples were extracted by vortex for 2 min after adding a piece of ceramic homogenizer in the tube. 1 g of sodium chloride and 4 g of anhydrous magnesium sulfate were added. The sample was hand-shaken again for 30 s. After centrifugation at 5000 rpm for 5 min, 0.2 mL of the top layer ACN was 5x diluted using ACN, then vortexed for 30 s. Finally, the tubes were filtered through a 0.22 µm nylon syringe filter for LC–MS/MS analysis.

### LC–MS/MS

**Fig. 2:**
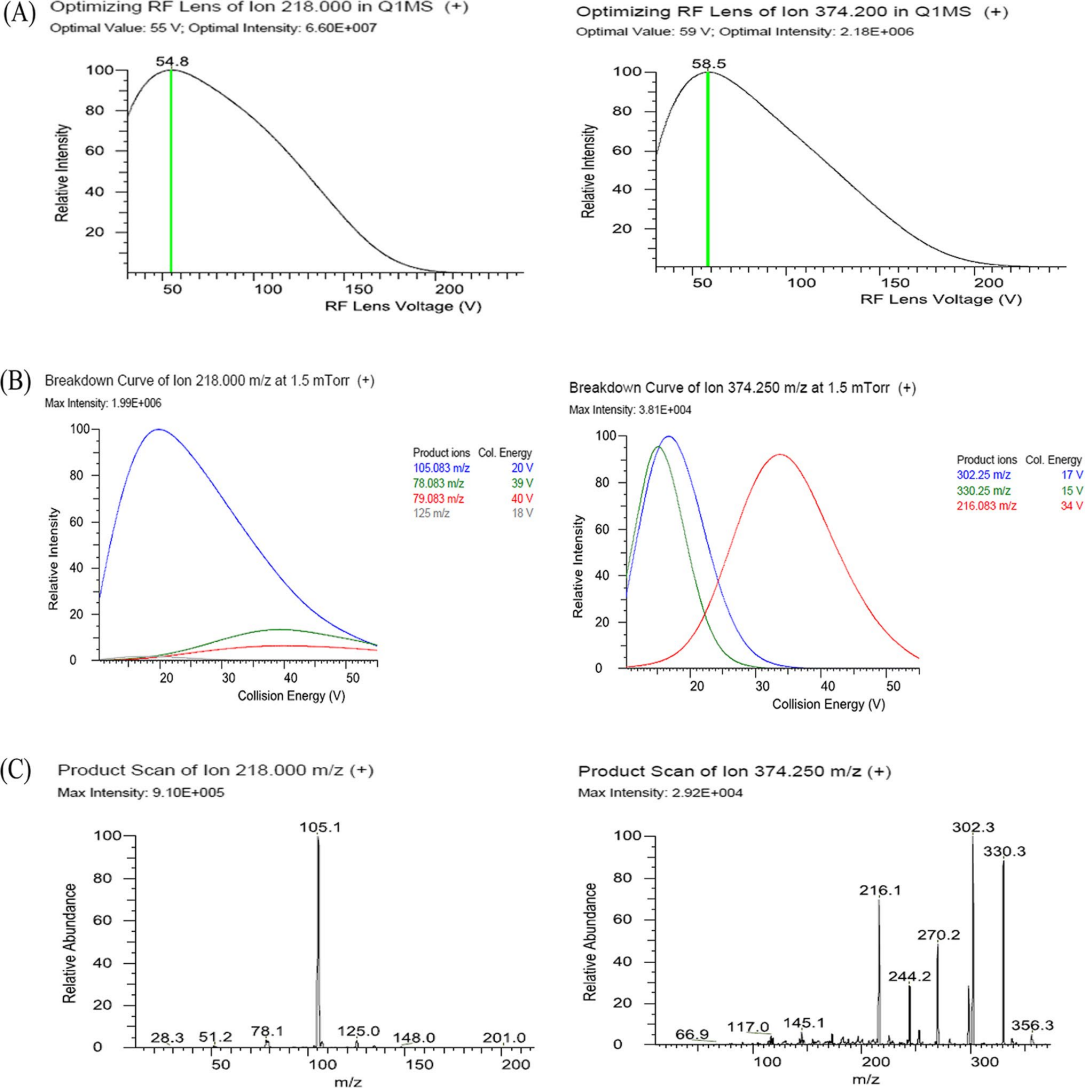
Optimizing Rf Lens (**A**), breakdown curve at 1.5 mTorr ( +) (**B**) and product scan (**C**) of pymetrozine (m/z 218) and spirotetramat (374.2 m/z)

A Dionex Ultimate™ 3000 RS UHPLC^+^ focused system separation module Liquid Chromatograph (LC) system (Thermo Fisher Scientific, Austin, TX, USA) in combination with TSQ Altis triple quadrupole mass spectrometer (MS/MS) was used to perform the LC–MS/MS analysis. The chromatographic separation was performed on the Accucore RP-MS C18 column (100 × 2.1 mm, 2.5 µm film thickness; Thermo Scientific, Lithuania) at 40 °C, with an injection volume of 1 µL. The mobile phase consisted of water/acetonitrile (30/70. v/v) with 0.1% formic acid at a total run time of 7 min. The pesticide detection was performed using the multiple reaction monitoring (MRM) mode. The optimal MRM transitions, collision energies (CE), and radio frequencies (RF) of S-lens were optimized using a standard solution of 0.5 mg/L in 50/50 MeOH/H_2_O with 0.1% formic acid at a constant flow rate of 0.3 mL/min and injection volume of 5 µL in an infusion mode. The electrospray ionization was operated in a positive mode (ESI^+^). The capillary ion spray voltage was 3800 V, the ion source temperature was 325 °C. The sheath and Aux gas pressure were 40 and 10 Arb, respectively. Trace Finder software (version 4.1) packages were applied to acquire and process the data obtained. Under these conditions, the retention times of pymetrozine and spirotetramat were 0.71 and 1.89 min, respectively. The specific MS/MS parameters are given in Table 1 and Fig. 2.

**Table 1:** MS/MS parameters and retention times for determination of pymetrozine and spirotetramat

Analyte	Precursor ion [M + H]^+^	Product ions (m/z)	Collision energy (V)	RF lens (V)	Rt (min.)
Pymetrozine	218	78.08	39.3	55	0.71
	218	105.08	19.8	55	
Spirotetramat	374.2	302.2	16.7	59	1.89
	374.2	330.2	15.1	59

### Method validation

Method performance was validated in terms of linearity, specificity, matrix effect (ME), accuracy and precision, and limit of quantification (LOQ), according to SANTE/12682/2019 guidelines (SANTE 2021). Linearity was assessed through the coefficient of determination (R^2^), residuals, and factors (RF) derived from the constructed external calibration curves. The constructed calibration curves that prepared in the blank sample, and that prepared in acetonitrile, were used to determine the percentage of matrix effect (%ME) as follows:

1 $$\mathrm{ME}\left(\mathrm{\%}\right)=\left(\mathrm{SMMC}-\mathrm{SSC}/\mathrm{SSC}\right)*100$$

where ME is the matrix effect, and SMMC and SSC are the slopes of the calibration curves in the matrix and pure solvent, respectively. A ME% of a positive value indicated that the matrix enhanced the analytical response, and a negative value showed that the matrix suppressed the analytical response. Blank green bean samples were analyzed to check the specificity of the method by observing if peaks occurred at or around the same retention time of the target analyte. The accuracy and precisions were estimated in recovery (%) and relative standard deviation (RSD, %), respectively. Blank green bean samples were spiked at four concentration levels of 0.005, 0.01, 0.1, and 1 mg/kg to confirm the method validity in the same day (intra-day repeatability) and three different days (inter-days repeatability). The LOQ was defined as the lowest spiked level achieving an acceptable recovery of 70–120% and precision of < 20%.

### Calculations

#### Dissipation and terminal residue

The degradation rates of pymetrozine and spirotetramat was calculated using a first-order kinetic model illustrated by Eq. (2)

2 $${\mathrm{C}}_{t}={\mathrm{C}}_{0}{\mathrm{e}}^{-\mathrm{kt}}$$

where C_*t*_ (mg/kg) is the residual levels of pymetrozine or spirotetramat at time t (days), C_*0*_ (mg/ kg) is the initial deposits. K is the first-order rate constant (day^−1^) obtained from the C_*0*_/C_*t*_ and t curve by regression analysis. The half-life (T_1/2_) is the time taken for a certain amount of pesticide to be reduced by 50%. The T_1/2_ was calculated by Hoskins’ formula (Eq. 3) (Hoskins 1961; Liang et al. 2011)**.** The safe pre-harvest interval (PHI) was computed using Eq. 4.

3 $${\mathrm{T}}_{1/2}=\mathrm{ln}2/\mathrm{k}$$

4 $$\mathrm{PHI}=\mathrm{Ln}\left(\mathrm{MRL}/{\mathrm{C}}_{0}\right)\mathrm{K}$$

#### Dietary intake risk assessment

The risks that may occur as a result of long-term dietary intake of the contaminated green beans with pymetrozine or spirotetramat was assessed using Eq. 5 and Eq. 6 (Malhat and Abdallah 2019).

5 $$\mathrm{NEDI}=\sum (\mathrm{STMRi}\times \mathrm{Fi})/\mathrm{bw}$$

The NEDI (mg/kg.bw/day) is the national estimated daily intake of the tested pesticide based on the Egyptian dietary intake. STMRi (mg/kg) is the supervised trials median residue value obtained from the field trials. Where Fi represents the consumption of green beans by the general population, and bw (kg) is the average body weight of adults (60 kg).

The risk quotient (RQ) was determined by Eq. 6

6 $$\mathrm{RQc}=\mathrm{NEDI}/\mathrm{ADI}\times 100$$

The ADI is the acceptable daily intake of pymetrozine (0.03 mg/kg.bw/day) (EFSA 2012) and spirotetramat (0.05 mg/kg.bw/day) (EFSA 2017).

## Results and discussion

### Method performance

**Fig. 3:**
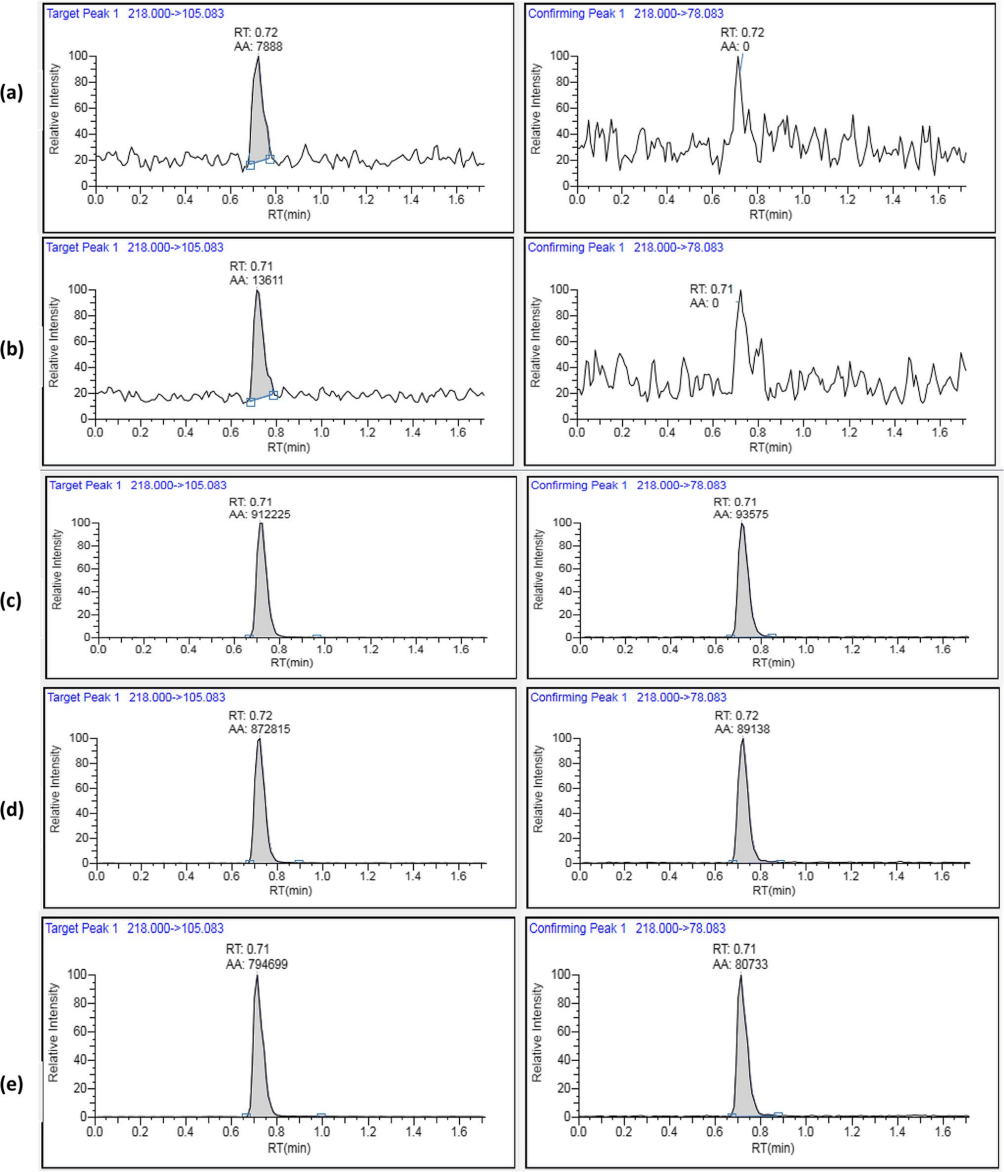
LC/MS/MS representative chromatograms of pure solvent (**a**), blank sample extract (**b**), pymetrozine standard in pure solvent (10 µg/l) (**c**), fortified sample extract (50 µg/kg) (**d**), spiked sample at 50 µg/kg (**e**)

**Fig. 4:**
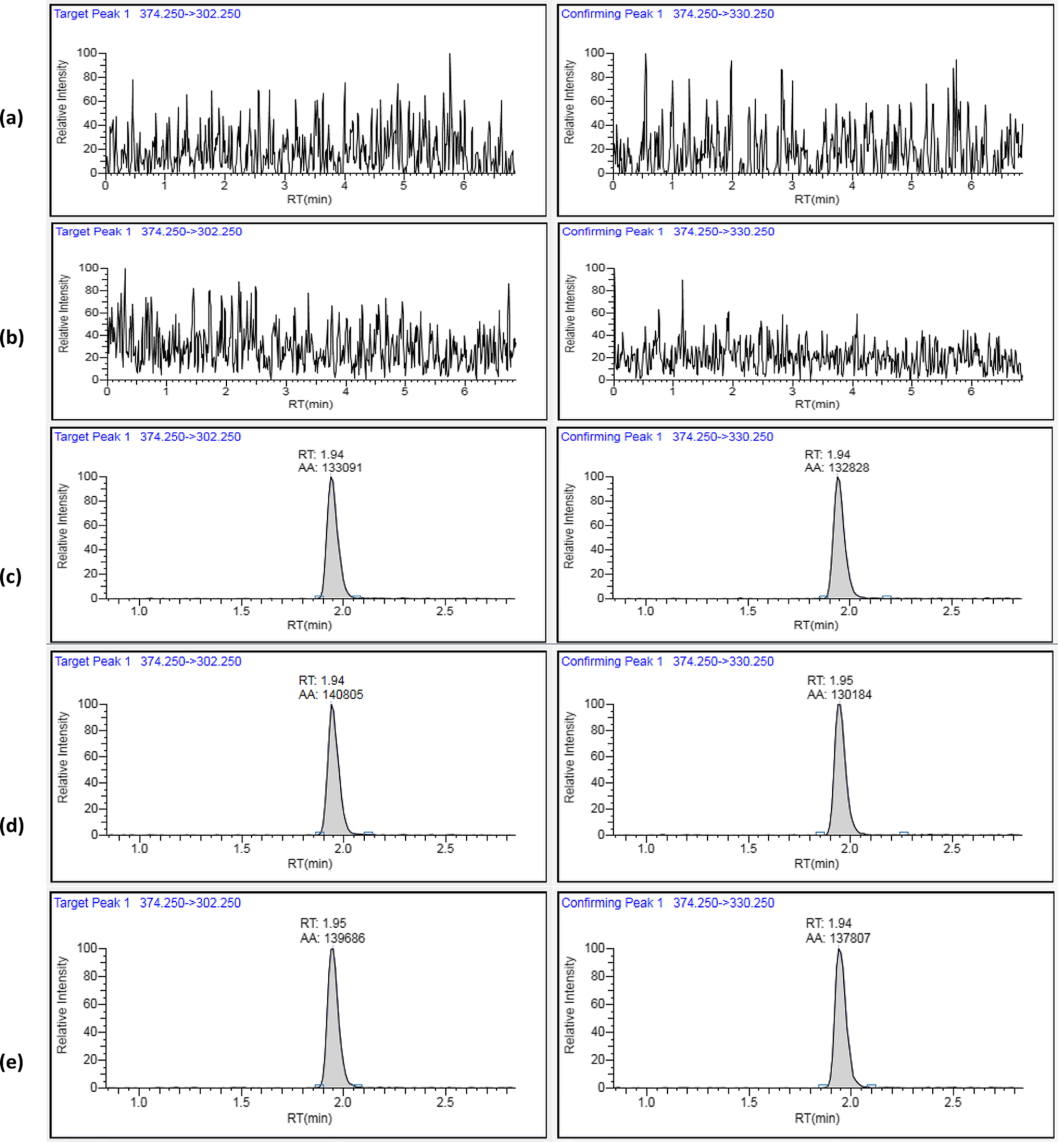
LC/MS/MS representative chromatograms of pure solvent (**a**), blank sample extract (**b**), spirotetramat standard in pure solvent (10 µg/l) (**c**), fortified sample extract (50 µg/kg) (**d**), spiked sample at 50 µg/kg (**e**)

To validate the specificity of the developed method, a representative blank sample of green beans was analyzed in triplicates to confirm that no matrix interfering peaks appears at the retention time of the target pesticides. Figures 3 and 4 showed that no interfering peaks appeared at or around the retention time of pymetrozine and spirotetramat, indicating that the method is specific. The selectivity of the method was confirmed by the identical retention time of pymetrozine and spirotetramat in the solvent and the matrix samples. The chromatograms of pymetrozine and spirotetramat resolved well in the solvent, blank, and fortified samples (Figs. 3 and 4).

Matrix-matched calibration curves of pymetrozine and spirotetramat were plotted for eight concentrations (0.001, 0.002, 0.005, 0.01, 0.025, 0.05, 0.1, and 0.2 μg/g). The calibration curves were linear with a correlation coefficient of r > 0.998, and response factors of ˂ 20%. The regression equations of SMMC were used for analytes quantification. The matrix effects of green beans were obtained using Eq. 1. Results showed that matrix of the green bean samples caused the suppression of pymetrozine responses with an ME equals to16.2%, while enhancement of spirotetramat responses were observed with an ME of 6.75%. To account for this effect this study used matrix-matched standard solutions to obtain more precise data (Table 2). The ME in UPLC-MS/MS is a consequence of the competition between the analyte and the complex matrix of the sample during ionization process in the electrospray ion source (Taylor 2005).

**Table 2:** Linearity range, Slope, Intercept, correlation coefficient(R^2^), residuals (%), response factor (RF) (%), matrix effect (%), and LOQ (mg/kg) of pymetrozine and spirotetramat in acetonitrile and green beans matrix

Analyte	Matrix	Range (µg/L)	Slope	Intercept	R^2^	Residuals (%)	RF (%)	ME (%)	LOQ (µg/kg)
Pymetrozine	Acetonitrile	2–100	7.945e^5^	3.586e^4^	0.9992	-4.71 to 5.6	-3.8 to -11.8	-	-
	Green beans	2–100	6.653e^5^	1.311e^4^	0.9997	-4.14 to 2.03	-2.8 to -7.2	-16.2	10
Spirotetramat	Acetonitrile	1–200	1.407e^5^	2.8e^4^	0.9988	-0.01 to 5.5	-1.5 to -18.7	-	-
	Green beans	1–200	1.502e^5^	2.697e^4^	0.9993	0.08 to -9.3	-1.3 to -19.1	6.75	5

The LOQs of pymetrozine and spirotetramat were 0.01 and 0.005 mg/kg, respectively (Table 2). The LOQ of pymetrozine and spirotetramat was 70 and 400 times lower than the MRL set by the European Union Commission (EFSA 2017, 2012).

Table 3 shows method precision and trueness expressed as repeatability (RSD%) and recovery (accuracy) (SANTE 2021). The efficiency of the extraction method was validated based on the recovery results (Table 3). The recovery study was developed at four concentration levels of 0.005, 0.01, 0.1, and 2 mg/kg, using six consecutive extractions for each spiked level. The mean recoveries ranged between 88.4% and 93.7% with RSD_r_ less than 11.6% for pymetrozine, and ranged between 95.1% and 103.4% with RSDr less than 7.7% for spirotetramat. The inter-days recovery and RSD_R_ (n = 18) for the tested concentration levels ranged from 88.9 to 92.4% with RSD_R_ less than 14.4% for pymetrozine, and 91.7 to 97.1% with RSD_R_ less than 12.4% for spirotetramat. Recoveries at the different concentration levels of the tested pesticides in green beans samples were satisfactory and within the SANTE recovery limits (70% ≤ Recovery ≤ 120%) and repeatability (≤ 20%) for the samples (SANTE 2021). This specified the accuracy and reproducibility of the developed method.

**Table 3:** Mean recoveries and RSD for pymetrozine and spirotetramat in green beans matrix

Analyte	Spiked level (mg/kg)	Intra-day		Inter-days	
		% Recovery	RSD_r_^a^	% Recovery	RSD_R_^b^
Pymetrozine	0.005	-	-	-	-
	0.01	93.7	8.3	90.4	10.2
	0.1	88.4	5.5	92.4	12.8
	2	90.2	11.6	88.9	14.4
Spirotetramat	0.005	95.1	4.8	93.4	6.1
	0.01	103.4	6.9	97.1	12.4
	0.1	98.7	3.2	95.5	7.5
	2	96.1	7.7	91.7	9.2

^a^intra-day repeatability (n = 6 for each spiking level, on the same day)

^b^inter-days repeatability (n = 18 for each spiking level, on three different days, 7 days intervals)

### Dissipation

**Fig. 5:**
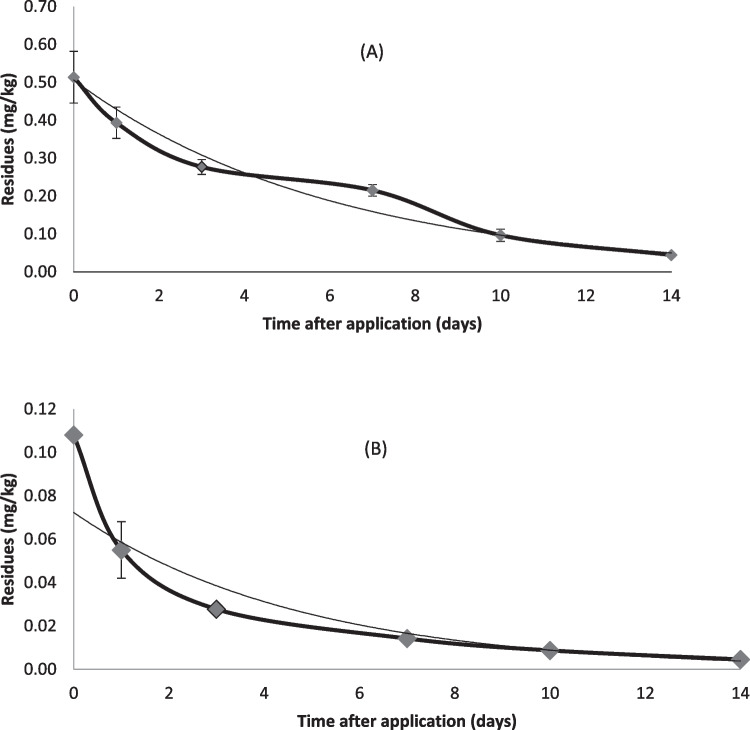
Dissipation curves for spirotetramat (**A**) and pymetrozine (**B**) after a single application to green bean grown in an open-field trials

Figure 5 shows the dissipation rate of pymetrozine and spirotetramat in green bean after one application. The first-order kinetic equation described the degradation rates of pymetrozine and spirotetramat with correlation coefficients of 0.953 and 0.970, respectively (Fig. 5). The initial concentrations of pymetrozine and spirotetramat of 0.108 mg kg^−1^ and 0.513 mg kg^−1^, respectively, decreased gradually as time lapsed (Fig. 5). After 14 days 95% of pymetrozine and 90% of spirotetramat were degraded. The growth dilution could be one of the reasons behind dissipation, while precipitation is not likely to be an important factor due to the low water solubility of spirotetramat (30 mg/L, 20 °C) and pymetrozine (270 mg/L, 20 °C).

In this study, the half-live of pymetrozine was 3.3 days, which is lower than those reported by Zhang et al. (2015) of 2.3—2.6 days in rice straw, Xu et al. (2021) of 6.79–11.36 days in strawberry and Abd-Alrahman and Kotb (2020) of 1.31 days in tomato. Meanwhile, the half-live of spirotetramat in the present study was 4.2 days which is less than values reported in other studies on other crops where it ranged between 4.4–8.1 days in citrus (Zhang et al. 2015), 12.4 days in pear (Xun et al. 2019).

The lower half-lives reported in this study compared to the studies of Zhang et al. (2015), Xun et al. (2019), Abd-Alrahman and Kotb (2020) and Xu et al. (2021), could be due to difference in some environmental factors during the experiment such as temperature, humidity, salinity and light intensity. These abiotic factors have major effects on plants metabolic and catalytic activities which may affect the dissipation of pesticides (Fahad et al. 2017, 2015b, 2015a). Furthermore, the differences in growth rate and chemical constituents between the different crops could have an effect on the half-lives of pesticides (Saber et al. 2020).

### Terminal residues

The terminal residues of pymetrozine and spirotetramat in green bean 3, 7 and 14 days after the last application are shown in Table 4. The residues of the two pesticides decreased by time whether were applied two or three times at the two tested concentrations (Table 4).

**Table 4:** Terminal residues of spirotetramat and pymetrozine in green beans

Dosage (g a.i/ha)	Number of times sprayed	Days after spraying	Terminal residues (mg/kg) ± SD	
			Spirotetramat	Pymetrozine
100	2	3	0.238 ± 0.048	0.175 ± 0.013
		7	0.153 ± 0.022	0.073 ± 0.021
		14	0.046 ± 0.014	0.015 ± 0.001
	3	3	0.496 ± 0.071	0.292 ± 0.019
		7	0.176 ± 0.038	0.155 ± 0.039
		14	0.042 ± 0.006	0.071 ± 0.007
200	2	3	0.427 ± 0.059	0.384 ± 0.044
		7	0.16 ± 0.066	0.144 ± 0.001
		14	0.047 ± 0.029	0.081 ± 0.003
	3	3	0.725 ± 0.095	0.537 ± 0.023
		7	0.148 ± 0.018	0.244 ± 0.051
		14	0.043 ± 0.02	0.018 ± 0.007

The concentrations of pymetrozine 3 and 7 days after application were higher when applied at double the recommended dose compared to applying the recommended dose. However, after 14 days the residues of pymetrozine decreased, reaching 0.081 ± 0.003 and 0.018 ± 0.007 mg kg^−1^ at double the recommended dose and the recommended dose, respectively. When spirotetramat was applied two and three times at recommended dose and twice the recommended dose based on PHI (14 days), residues of spirotetramat were 0.042–0.047 mg kg^−1^ and 0.043–0.047 mg kg^−1^, respectively. The European MRL for both pesticides, spirotetramat and pymetrozine in fresh beans with pods is 2 mg kg^−1^ (EFSA 2021, 2012). In the present study, the terminal residues at all studied time intervals and concentrations were far below the European MRL, which indicated good agricultural practice complying with consumer safety and product international trading (Saber et al. 2020).

### Risk assessment

The potential risk to humans associated to the consumption of green beans with residues of pymetrozine and spirotetramat at the levels reported in this study was assessed using the RQ (Table 5). Results showed that the RQ values of pymetrozine and spirotetramat in green bean were all below 100% at the recommended dosage and also at twice recommended dosage applied two and three times. This indicated that the residue levels of pymetrozine and spirotetramat in green bean do not pose hazardous effects to consumers at the studied concentrations.

**Table 5:** Dietary intake and risk quotient (%) through consumption of green beans treated with spirotetramat and pymetrozine

Dosage (g a.i/ha)	Number of times sprayed	Days after spraying	Spirotetramat		Pymetrozine	
			NEDI (mg/kg bw)	RQ (%)	NEDI (mg/kg bw)	RQ (%)
100	2	3	1.94E-03	3.887	1.43E-03	4.764
		7	1.25E-03	2.499	5.99E-04	1.996
		14	3.81E-04	0.762	1.20E-04	0.399
	3	3	4.06E-03	8.112	2.38E-03	7.940
		7	1.44E-03	2.880	1.27E-03	4.229
		14	3.40E-04	0.681	5.80E-04	1.933
200	2	3	3.48E-03	6.969	3.14E-03	10.462
		7	1.31E-03	2.613	1.18E-03	3.920
		14	3.87E-04	0.773	6.64E-04	2.215
	3	3	5.92E-03	11.836	4.38E-03	14.609
		7	1.21E-03	2.412	1.99E-03	6.633
		14	3.48E-04	0.697	1.50E-04	0.499

## Conclusions

The present study has validated a QuEChERS extraction method for the residual analysis of pymetrozine and spirotetramat in green bean using LC–MS/MS. Field experiments were conducted to study the dissipation dynamics and terminal residues of pymetrozine and spirotetramat in green bean. In addition, a risk assessment study was conducted on the dietary intake of pymetrozine and spirotetramat in green bean based on their residues after field trials. In this study, the average recovery of pymetrozine and spirotetramat in green bean was ˃88.4%, with precision of ˂ 14.4% at fortification levels of 0.005, 0.01, 0.1 and 2 mg/kg. The LOQs of pymetrozine and spirotetramat were 0.005 mg/kg and 0.01 mg/kg, respectively. Field experiments showed that the degradation of pymetrozine and spirotetramat in green bean followed a first order reaction kinetic equation. Half-lives of pymetrozine and spirotetramat were 4.2 and 3.3 days respectively. The RQ values were far below 100%, indicating that pymetrozine and spirotetramat at the residue levels reported in the field experiments, do not pose risk to consumers. The present study has provided a scientific basis for the safe application of pymetrozine and spirotetramat in green bean under Egyptian field conditions.

## Data Availability

Not applicable.
